# Thermodynamic Factors That Drive Sequence-Specific DNA Binding of Designed, Synthetic Minor Groove Binding Agents

**DOI:** 10.3390/life12050681

**Published:** 2022-05-04

**Authors:** Ananya Paul, Abdelbasset A. Farahat, David W. Boykin, W. David Wilson

**Affiliations:** 1 Department of Chemistry and Center for Diagnostics and Therapeutics, Georgia State University, Atlanta, GA 30303, USA; apaul@gsu.edu (A.P.); a4f2002@mans.edu.eg (A.A.F.); dboykin@gsu.edu (D.W.B.); 2 Department of Pharmaceutical Organic Chemistry, Faculty of Pharmacy, Mansoura University, Mansoura 35516, Egypt

**Keywords:** DNA minor groove binder, mixed base-pair DNA sequences, sequence selectivity, ligand–DNA complex thermodynamic, molecular curvature, heterocyclic diamidine, biosensor, calorimetry

## Abstract

Ken Breslauer began studies on the thermodynamics of small cationic molecules binding in the DNA minor groove over 30 years ago, and the studies reported here are an extension of those ground-breaking reports. The goals of this report are to develop a detailed understanding of the binding thermodynamics of pyridine-based sequence-specific minor groove binders that have different terminal cationic groups. We apply biosensor-surface plasmon resonance and ITC methods to extend the understanding of minor groove binders in two directions: (i) by using designed, heterocyclic dicationic minor groove binders that can incorporate a G•C base pair (bp), with flanking AT base pairs, into their DNA recognition site, and bind to DNA sequences specifically; and (ii) by using a range of flanking AT sequences to better define molecular recognition of the minor groove. A G•C bp in the DNA recognition site causes a generally more negative binding enthalpy than with most previously used pure AT binding sites. The binding is enthalpy-driven at 25 °C and above. The flanking AT sequences also have a large effect on the binding energetics with the -AAAGTTT- site having the strongest affinity. As a result of these studies, we now have a much better understanding of the effects of the DNA sequence and compound structure on the molecular recognition and thermodynamics of minor groove complexes.

## 1. Introduction

In the 1980s, Ken Breslauer and some excellent coworkers initiated a series of fundamental studies on the thermodynamics of small-molecule, minor groove agents, and intercalators, binding to different DNA sequences [1,2,3,4,5,6,7]. Among other techniques, they used batch calorimetry in ground-breaking investigations of the DNA complexes of these compounds. They established methods for these types of studies that have continued to influence thermodynamic analysis of small-molecule-DNA binding to this day, including the work reported here. They introduced concepts and methods such as entropy-enthalpy compensation that masked driving forces when looking at free energy alone [5]. They used structural studies to bring an understanding of the structural and solution properties that influence the thermodynamics of DNA complex formation [4]. In these studies, they began to develop a microscopic understanding of the experimental macroscopic thermodynamic results. They started the research with state-of-the-art batch calorimeters, which, from personal experience with one of my early colleagues, Harry Hopkins, require a thermodynamics artist’s touch to bring forth beautiful thermodynamics pictures. They later moved to much-improved titration calorimeters from MicroCal but continued with detailed thermodynamics studies of DNA interactions that now are going on around the world. It is a pleasure to write this paper in honor of Ken’s 75 birthday.

**Figure 1:**
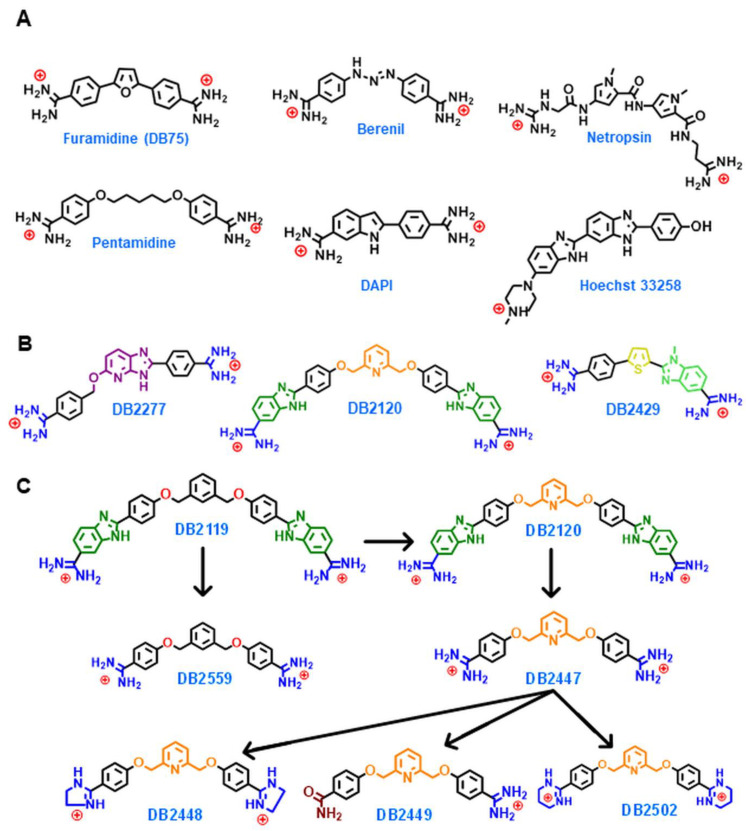
(**A**) Structures of classical AT-specific DNA minor groove binders; (**B**) structures of three very different designed mixed-sequence DNA minor groove binders; (**C**) schematic representation of the development of AT-specific DNA binding compounds from DB2119 to DB2559 and from mixed DNA sequence-specific compounds DB2120 to DB2447 and the analogs for this study. Color schemes used in this figure denote different functional groups.

The Breslauer and other laboratories began studies with available, classical minor groove binders from polyamides, such as netropsin, to other types of cationic heterocycles, such as berenil and Hoechst dyes, which were all specific for binding to AT DNA sequences (Figure 1A) [1,2,3,4,5,6,7,8]. These are all uniformly concave-shaped compounds that fit snugly into the minor groove in A-tract sequences and have groups that can H-bond with the N3 of dA or O2 of dT at the floor of the groove.

In addition to forming an H-bond with the cytosine C=O in the minor groove, the 2-amino group of G projects an -N-H into the groove and presents a steric block to classical AT-specific minor groove binders [9]. In spite of the many successes with these early minor groove binders, the lack of a broad selection of sequence-specific compounds was a limitation for expanded applications. Crystal structures of DNA and complexes suggested that compounds with H-bond acceptor groups could bind to the free G-NH in the minor groove to give GC binding specificity [9,10,11,12,13,14]. This concept was successful with polyamides but has not been broadly applied to the other minor groove binders such as those listed above (Figure 1).

As heterocyclic amidine minor groove binders such as 4′,6-diamidino-2-phenylindole (DAPI) are widely used as cellular nuclear stains and others such as pentamidine, furamidine, and berenil (Figure 1A) have been used or tested in humans and/or animals for therapeutic applications [15,16,17,18,19], we have used the heterocyclic amidine template as the platform for incorporation of single hydrogen bond-accepting groups in generating the first set of compounds with increased specificity. The goal with these first compounds was to recognize a GC base pair in an AT sequence. In this way, it would be possible to create modules that could be combined to give broad DNA sequence recognition. Successful compounds with azabenzimidazole, N-alkylbenzimidazole, and pyridine H-bond acceptor groups for G recognition were created (Figure 1B) [20,21,22,23,24,25]. Extensive elaborations of the N-alkylbenzimidazole module have significantly improved the affinity and selectivity of that module. With a thiophene adjacent to the imidazole of N-alkylbenzimidazole, a preorganized structure for minor groove recognition was created [25,26,27,28].

**Figure 2:**
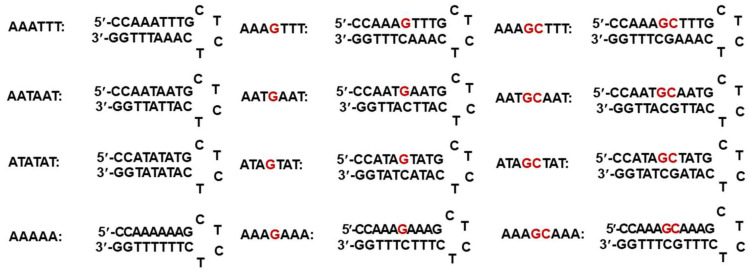
The DNA sequences used in this study; DNA sequences used for Surface Plasmon Resonance (SPR) studies were labeled with 5′-biotin.

The original pyridyl-linked amidine-benzimidazole-phenyl compound, DB2120, binds strongly with the single G•C bp-containing -A4GT4- target sequence with a *K*_D_ < 0.1 nM. DB2120, however, has poor solution properties and aggregation difficulties even at low concentrations under standard experimental conditions for DNA complexes. Because of its size and AT binding benzimidazole units, it also has less selectivity than optimum for use in most applications. To increase the possible uses of the pyridine series of compounds, it is, thus, essential to develop smaller molecules that will have better solution and sequence recognition characteristics. Hence, in the studies reported here, in-depth experiments were conducted with 12 primary DNA hairpin duplex sequences (Figure 2) four of which contain pure AT sequences in their recognition sites (AAAAAA, AAATTT, AATAAT, and ATATAT), four have mixed AT and single GC bp DNA sequences (AAAGAAA, AAAGTTT, AATGAAT, and ATAGTAT), and four have AT sequences with two G•C bps (AAAGCAAA, AAAGCTTT, AATGCAAT, and ATAGCTAT). Three pyridine compounds that are smaller than DB2120, as well as a phenyl compound, were synthesized with the smaller compound design ideas (Figure 1C). The smaller compounds are easier to synthesize, have improved solution properties, and have strong and specific binding to DNA sequences with a single G•C bp. The results with these new pyridine and phenyl compounds are reported here.

## 2. Compound Design

The goal with these compounds was to make a series of DB2120 analogs of reduced size but better solution properties and with strong and selective binding to target single G•C bp-containing sequences [23,24]. The compounds would retain the DB2120 core structure but with different terminal cationic groups. Dications with amidine, imidazoline, and tetrahydropyrimidine were successfully prepared along with a monocation with terminal amidine and amide groups (Figure 1C). As a control, an amidine analog with a central phenyl ring that cannot form the H-bond required for GC specificity was also prepared. By using our relative curvature determination method [28] (Results), the compounds were found to have an optimum curvature for the minor groove. Results with these five compounds and the target and control DNA sequences described above (Figure 2) are reported here. For complete studies, target sequences have a single G•C bp in an AT sequence context, while the control sequences have no G•C bp or a two G•C bp insert.

## 3. Results

### 3.1. Compound Synthesis

**Scheme 1:**
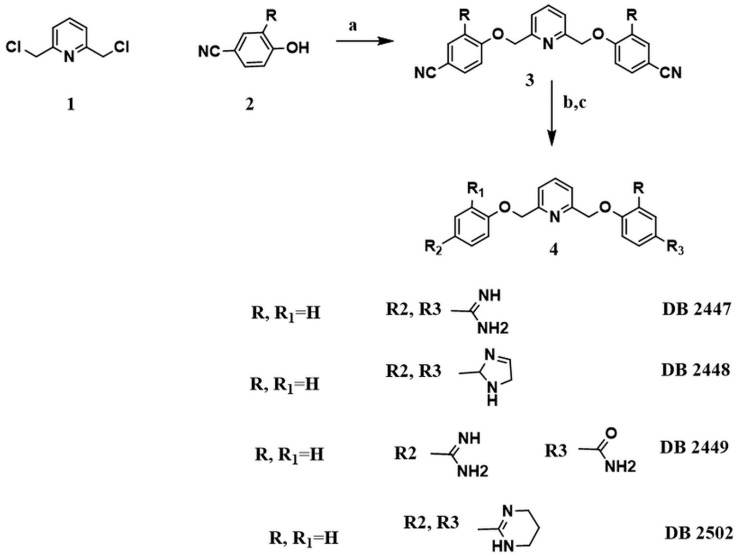
Reagents and conditions: (**a**) K_2_CO_3_, DMF, 45 °C, 4 h; (**b**) ethanol-HCl, 0 °C—r.t.; (**c**) ethanol, ammonia or amine, r.t or reflux.

Scheme 1 describes the synthesis of the final amidines **4**. Cyanophenol derivatives **2** were allowed to react with bis(chloromethyl)pyridine **1** in anhydrous dimethylformamide in the presence of potassium carbonate as a base. The formed bisnitriles were converted to the final amidines by applying Pinner reaction conditions [29,30], where the bisnitriles were converted to the intermediate imidate ester hydrochloride by stirring in dry ethanolic HCl. The formed imidate ester was converted to the corresponding amidine by stirring in dry ethanolic ammonia. Finally, the amidines were purified by conversion to the free base using sodium hydroxide and then formation of the hydrochloride salt by stirring in ethanolic HCl. The characterization of the final compounds have described in Experimental Methods.

### 3.2. DNA Thermal Melting (ΔT_m_): Screening for Relative Affinity and Sequence Selectivity for Target and Control DNA Binding

**Figure 3:**
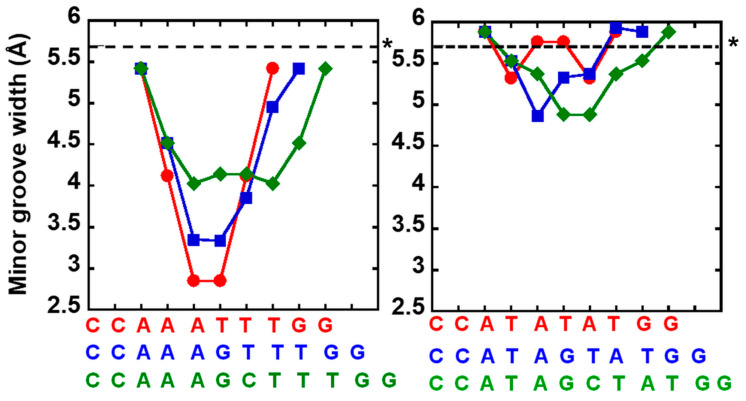
Minor groove width vs. target DNA sequences calculated from the online algorithm of Rohs and coworkers (*Nucleic Acids Res.* **2013**, *4*, W56-62). * indicates the minor groove width of standard *B*-form DNA. Groove width gives the perpendicular separation of helix strands drawn through phosphate groups, diminished by 5.8 Å to account for van der Waals radii of phosphate groups.

Changes in DNA thermal melting temperature (Δ*T*_m_) provide an initial ranking of compounds for binding affinity and relative sequence specificity with the target and control DNA sequences [31,32]. The binding affinities of the pyridyl-centered heterocyclic cations were tested with pure AT sequences, which are the primary sites of most known minor groove binders from netropsin to furamidine (Figure 1A) [33,34,35]. The target DNAs of primary interest for the compounds in this research have a single G•C bp with flanking AT sequences. The *T*_m_ experiments were carried out in the presence of nonalternating and alternating AT, and mixed flanking sequences, AAA-TTT, ATA-TAT, AAT-AAT, and AAA-AAA, Figure 2, where the dashes indicate zero, one, or two G•C bps. These are useful test sequences as each flanking AT sequence has different properties, including variations in the minor groove positions of the H-bond acceptor groups on the A•T bp. The number of G•C bp also varies in these selected mixed DNA sequences, which also gives significant differences in minor groove width (Figure 3).

**Figure 4:**
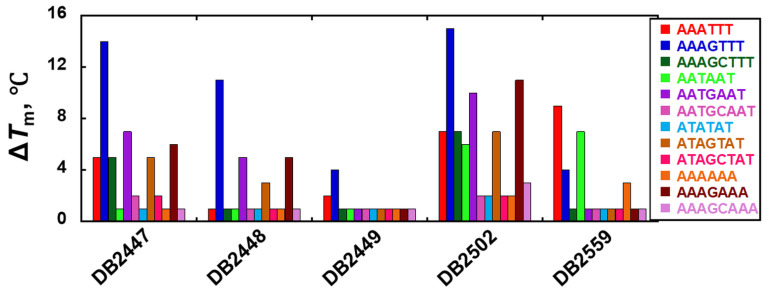
Comparison of relative binding affinities, Δ*T*_m_, °C (Δ*T*_m_ = *T*_m (the complex)_ − *T*_m (the free DNA)_) by thermal melting experiments of the designed heterocyclic amidine compounds with pure AT base-pair and mixed single G•C base-pair-containing DNA sequences. The listed values are for the 2:1 [ligand]/[DNA] ratio and an average of two independent experiments with a reproducibility of ±0.5 °C. Full DNA sequences are as described in Figure 2.

With AAA-TTT, a type of nonalternating AT sequence, DB2447, the direct truncated analog of the original pyridine, DB2120, resulted in an encouraging increase in the thermal stability of the single G•C bp-containing target site sequences (Figure 4 and Table S1). DB2447 also showed sequence selectivity, as expected from previous results and the compound design approach. The compound showed significantly lower thermal stability increases for the two G•C bps and all AT-containing sequences (Figure 4 and Table S1).

Two analogs of DB2447 with terminal 4,5-dihydro-1-H-imidazole (*Im*) (DB2448) and 1,4,5,6-tetrahydro pyrimidine (*THP*) (DB2502) were synthesized and tested to determine how the molecular size and chemistry of terminal dications affect the sequence binding affinity and selectivity. The *Im* compound binds to a single G•C bp DNA somewhat more weakly than DB2447 but with significantly improved selectivity (Figure 4 and Table S1). The *THP* compound DB2502 binds to the single G•C bp target slightly more strongly than DB2447 and with similar selectivity. These results indicate that the terminal *Im* and *THP* analogs are useful additions to expand the chemical space of the parent, DB2447. The three compounds can be especially useful in biological test applications where their uptake into different cell types can be very different. In cellular applications where strong specificity is needed, the Im analog may be preferred. The compound DB2449, with one amidine replaced with an amide, has quite low Δ*T*_m_ values with all of the tested DNAs (Figure 4 and Table S1).

In addition to the compound structure and properties, the DNA sequence has a major effect on minor groove binding. The alternating DNAs ATA-ATA have a significant drop in Δ*T*_m_ relative to the target, AAA-TTT sequences with all compounds. This is expected based on the wider Minor Groove (MG) of the alternating sequence (Figure 3 and Figure 4 and Table S1) [36,37]. The sequence with a pure A-tract AAA-AAA also has a surprisingly lower Δ*T*_m_ relative to the target, AAA-TTT sequences. The AAT-AAT sequences have an intermediate Δ*T*_m_ relative to the target, AAA-TTT sequences. In summary, the -AAAGTTT- sequence has the best Δ*T*_m_ of all of the flanking AT sequence variations. This can easily be seen in Figure 4, where the three pyridyl-diamidines with each DNA sequence group have the highest peaks in the histogram set. With DB2559, the -AAATTT- and -AATAAT- sequences have the highest Δ*T*_ms_ as expected for a central phenyl. Analysis of the minor groove widths of the different single G•C bp sequences showed that of all sequences, -AAAGTTT- has the most narrow minor groove and is most appropriate for binding compounds such as DB2447 with a connected aromatic system of approximately 3.4 A in width (Figure 3). The weakest binding is seen with the -ATAGTAT- sequence and it has the widest DNA minor groove, basically the same as a standard B-form minor groove. The groove widths for the -AAAGTTT- and -ATAGTAT are shown in Figure 3 for reference.

### 3.3. Biosensor-Surface Plasmon Resonance (SPR): High-Resolution Evaluation of Binding Affinity, Kinetics, Stoichiometry, and Cooperativity

**Figure 5:**
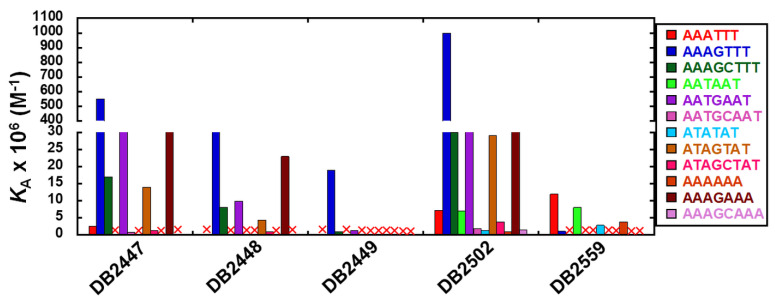
Comparison of equilibrium binding constants (*K*_A_, M^−1^) of phenyl and pyridine analogs with pure AT and mixed single G•C base-pair-containing DNA sequences. “**X**” represents no measurable *K*_A_ under our experimental conditions. The listed binding affinities are an average of two independent experiments carried out with two different sensor chips, and the values are reproducible within a 10% experimental error. Full DNA sequences are as described in Figure 2.

**Figure 6:**
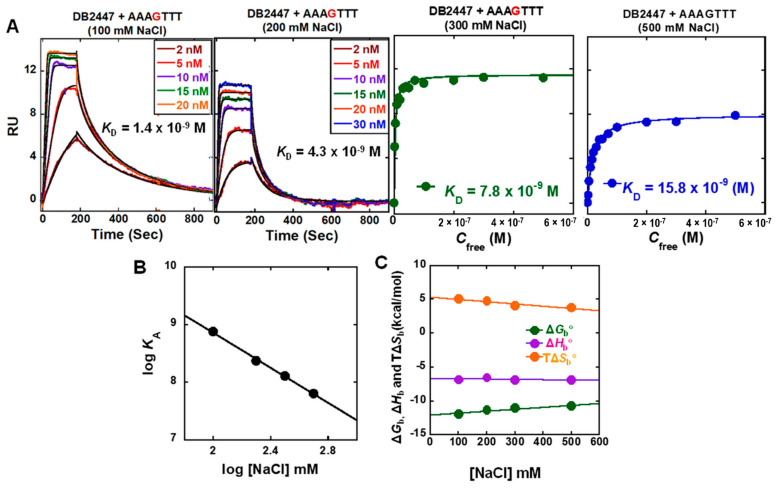
(**A**) SPR sensorgrams with kinetics fits and steady-state binding plots for DB2447 with the AAAGTTT DNA sequence at different salt concentrations; (**B**) salt dependence of *K*_A_ for DB2447 binding as determined by SPR. The *K*_A_ values were obtained by global kinetics (at two lower salt concentrations) and steady-state fits (at two higher salt concentrations); (**C**) plot of Δ*G*_b_°, Δ*H*_b_°, and TΔ*S*_b_° versus salt concentrations for DB2447 with AAAGTTT sequence at 25 °C. The listed binding affinities are standard thermodynamic values and are an average of two independent experiments carried out with two different sensor chips. The values are reproducible within a 10% experimental error; RU = Response Unit based on amounts of bound compounds on the immobilized DNA.

Biosensor-SPR methods provide a high-resolution, label-free way to quantitatively evaluate the binding affinity, selectivity, and stoichiometry of a set of compounds with a spectrum of immobilized DNAs [38,39,40]. The parent pyridyl-linked-phenyl-amidine, DB2447, binds strongly with AAAGTTT, and global kinetics fitting defined a single binding site with *K*_A_ = 5.5 × 10^8^ M^−1^ (*K*_D_ = 1.8 nM) at 0.1 M NaCl (Figure 5 and Figure 6, Table S2). The strong binding of DB2447 is the result of the rapid association that is at the instrumental limitation (*k*_a_ = ~4.4 × 10^7^ M^−1^s^−1^) and a comparatively slow dissociation rate constant (*k*_d_ = 7.8 × 10^−2^ s^−1^). DB2447 binds to the pure AT, -AAATTT-, sequence as a monomer complex with rapid dissociation and a 200-fold lower affinity compared to AAAGTTT. This result indicates that DB2447 maintains surprisingly high sequence selectivity for the single G•C bp sequence. The sensorgram of AAATTT shows an off-rate that is much faster, and complete dissociation from the complex occurs within the first few seconds of the dissociation phase (Figure S1). With the -AAAGCTTT- binding site, DB2447 shows a 30-fold weaker binding affinity than with -AAAGTTT- under the same experimental conditions (Figure 5 and Figure S1, Table S2). It appears that DB2447 can induce a somewhat favorable minor groove site in -AAAGCTTT- for binding, but the extra GC is a mismatch that reduces the binding affinity. The phenyl derivative, DB2559, with a simple -*N*- to -*CH* conversion, has a stronger binding for pure AT sequences, over ten times stronger than with the single G•C sequence, as with most classical minor grooves binders (Figure 5 and Figure S2, Table S2). This result is as expected for compounds without an H-bond acceptor group in a position to bind to the G-NH that faces into the minor groove.

The derivative, DB2448, with an imidazoline terminal group has a *K*_D_ value about half as strong as DB2447 for the single G•C bp sequence, but it has negligible binding to AAATTT under our experimental conditions, an impressive improvement in sequence selectivity (Figure 5 and Figure S3, Table S2). DB2502, on the other hand, with six-atom terminal cations has a *K*_D_ value that is about twice as strong as that of DB2447. Unfortunately, its *K*_D_ value for the pure AT sequence is about three times stronger than for DB2447. With the AAAGCTTT sequence, DB2448 has a *K*_D_ value over twice that of the DB2447 constant but the DB2502 value is below half of the DB2447 *K*_D_ value. The *Im* substitution thus has a promising increase in overall selectivity while the results with DB2502 are a disappointment in selectivity for this series (Figure 5 and Figure S4, Table S2). As with the *T*_m_ experiments, DB2449, the monocation, has relatively weak binding to all tested DNA sequences except -AAAGTTT with a *K*_D_ of 52 nM (Figure 5 and Figure S5, Table S2). This compound has very different solution properties relative to all other compounds and may be an advantage in cell studies.

With the other three sets of flanking sequences, the single G•C bp sequences always have the strongest binding (Figure 5 and Figures S1–S5, Table S2), as with -AAAGTTT-, but the binding is weaker than with -AAAGTTT. With -AAAGAAA- and AATGAAT, the binding with DB2447 is about a factor of ten weaker than with -AAAGTTT-. With the fully alternating sequence -ATAGTAT-, the binding is reduced by close to a factor of 40. Similar reductions in affinity are seen with the other compounds and these DNA sequences (Figure 5, Table S2). With the Figure 5 histograms, the single G•C set of sequences have the highest plot in each set of DNAs. It should also be noted, however, that the GC sequences have a stronger-than-expected binding.

### 3.4. Molecular Curvature Determination

**Figure 7:**
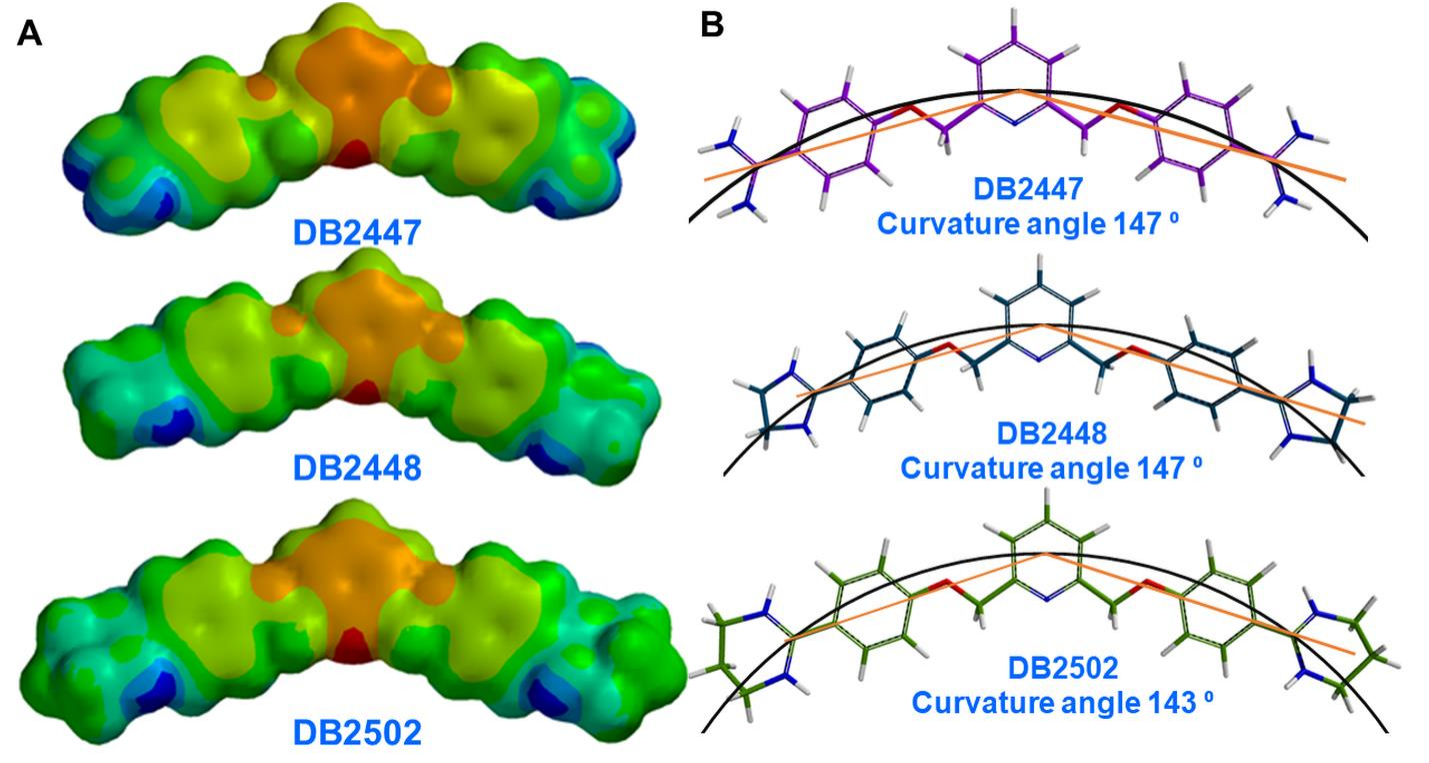
(**A**) Energy-minimized structures of DB2447, DB2448, and DB2502 at B3LYP/6-31G* (p,d) level of theory. In the electrostatic potential maps, red indicates high electronegativity and blue indicates electron-deficient/positively charged regions. (**B**) Molecular curvatures for DB2447, DB2448, and DB2502. Black circles represent reference circles and orange lines represent two straight lines drawn from the circle point at the center of the molecule to the amidine carbons.

Molecular curvature plays a crucial role in sequence-selective DNA minor groove recognition in conjunction with DNA minor groove binders’ molecular functionality and stacking surface. Correct curvature is important for strong H-bonding interaction, charge interactions, and van der Waals stacking in the groove of the DNA. Our previous report [28] stated a graphical approach method to determine relative molecular curvature values for minor groove binding compounds. In this approach, the diamidines compounds are energy-minimized in the SPARTAN software package using the DFT/B3LYP theory with the 6-31+G* basis set. The compounds are then matched up in a PowerPoint graphics package. A reference circle (black circle) is drawn through both amidine carbons, the center of the compound where the circle’s periphery passes through the center point of the individual molecular unit of the entire molecule, illustrated in DB2447, DB2448, and DB2502 (Figure 7). Two straight lines (orange) are drawn from the circle point at the center of the molecule to the amidine carbons. The midpoint of these two lines defines the comparative curvature value for the diamidines compound. The curvature values are 147° for DB2447, 147° for DB2448, and 143° for DB2502. The curvature analysis of a library of strong DNA minor groove binding compounds by this method offers a standardization value of around 140–145° curvature angle. DB2447 and its analogs also show a similar ° of curvature angle, which supports the strong binding affinity of these diamidines compounds.

### 3.5. Effect of Salt Concentration and Temperature on DB2447 Binding to Single G•C bp DNA Sequences

The *T*_m_, structure evaluation, and SPR binding results indicate that DB2447 has an optimized length, curvature, and flexibility for effective and selective recognition of a single G•C bp in an AT minor groove sequence. The SPR results also indicate that DB2447 has improved solution properties relative to DB2120, which allows us to explore the thermodynamic behavior of this compound with single G•C bp and other sequences in more detail. These results help to provide a fundamental understanding of the molecular basis for specific recognition of the DNA minor groove.

To evaluate the effect of ionic strength on DB2447 binding affinity with the -AAAGTTT- sequence, SPR experiments were carried out from 100 to 500 mM NaCl concentrations at 25 °C (Figure 6, Table 1). The equilibrium binding constants (*K*_A_) obtained either by global kinetics fits at low salt concentration or by steady-state fits at higher salt concentration are collected in Table 1 and Figure 6. Both theory and experiment suggest that the logarithm of the equilibrium binding constant *K*_A_ is a linear function of the logarithm of NaCl concentration for many organic cations binding to DNA [41,42]. For a typical DNA−cation complex, the equilibrium binding constant values decrease as the salt concentration increases with a slope that depends on the compound charge [42,43,44,45,46]. As seen in Figure 6, the log(*K*_A_) versus log[Na^+^] plot for DB2447 is linear with a slope of 1.6. The number of phosphate contacts (*Z*) between DB2447 and the AAAGTTT DNA sequence is predicted to be two and can be obtained in experiments from the slope/0.88, where 0.88 is the fraction of phosphate charge shielded by the total associated counterions. For the 11-base-pair synthetic oligomer AAAGTTT, the obtained *Z* is 1.8. These results indicate that the dicationic DB2447 releases 2 Na^+^ ions when binding to the DNA minor groove. The enthalpy change, Δ*H*_b_°, for binding is essentially constant with salt concentration, while for both Δ*G*_b_° and TΔ*S*_b_°, values decrease by one kilocalorie with the change in salt concentration from 100 to 500 mM NaCl.

**Figure 8:**
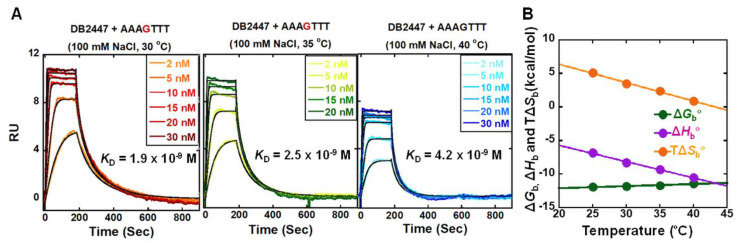
(**A**) SPR sensorgrams with kinetics fit for DB2447 with the AAAGTTT DNA sequence at different temperatures at 100 mM NaCl; (**B**) thermodynamic results, from Table 2 for binding of DB2447 to the AAAGTTT site at different temperatures. The listed binding affinities are standard thermodynamic values and binding affinities are an average of two independent experiments carried out with two different sensor chips, and the values are reproducible within a 10% experimental error; RU = Response Unit based on amounts of bound compounds on the immobilized DNA.

To obtain an additional understanding of the thermodynamic basis for DB2447 interactions with DNA, SPR experiments were conducted from 25 °C to 40 °C at 100 mM salt concentration. The SPR results reveal that temperature significantly affects the DB2447-DNA binding thermodynamics and kinetics, as shown in Figure 8 and Table 2. The ligand-DNA binding affinity decreases (*K*_A_) with experimental temperature. However, the temperature changes have a smaller effect on the Δ*G*_b_° as previously observed for other minor groove binders [42]. As can be seen in Figure 7, the enthalpy and entropy for binding have compensating decreases with increasing temperature. The TΔ*S*_b_° and Δ*H*_b_° values are similar at 20 °C but TΔ*S*_b_° approaches zero at 45 °C, and Δ*H*_b_° completely accounts for Δ*G*_b_°.

### 3.6. Isothermal Titration Calorimetry (ITC) of Complex Formation: Effects of Salt Concentration and Temperature 

Characterization of the full thermodynamics of cationic compound–DNA interactions is an essential component of any detailed analysis of DNA molecular recognition and rational drug design. Isothermal titration calorimetry (ITC) is the method of choice to analyze the energetic basis for the strong and selective binding of compounds with the DNA minor groove. It has been used with minor groove binders since the early studies of Ken Breslauer and colleagues and it is applied here to DB2447–DNA complexes. ITC provides a key component of the thermodynamic profile of ligand–DNA interactions by direct determination of the enthalpy (Δ*H*_b_°) of binding. With the enthalpy and Δ*G*_b_° from biosensor experiments, it is possible to calculate the entropy of binding (Δ*S*_b_) from the known thermodynamic relationships (Δ*G*_b_° = −RTln*K*_A_) and (Δ*G*_b_° = Δ*H*_b_° − TΔ*S*_b_). Thermodynamic profiles are valuable in drug design because they provide quantitative data on drug–DNA interactions that cannot be obtained directly by structural or computational methods [47,48,49,50,51]. They also provide valuable ideas about the compound-DNA-water components of complex formation [52].

In the experiments reported here, ITC was used to monitor the heat released upon the binding of DB2447 to the target binding sites. For strong binding compounds such as DB2447, ITC experiments require considerably higher concentrations than the compound *K*_D_ for DNA interactions. In cases such as this, the enthalpy of binding can be determined quite accurately in the presence of excess DNA in the calorimetry cell such that the compound is fully bound to the DNA. In this model-free approach, the Δ*H*_b_° is simply determined from the average of Δ*H*_b_° versus the binding ratio below saturation binding. The binding constant must then be determined at lower concentrations by an alternative method such as SPR or fluorescence methods.

**Figure 9:**
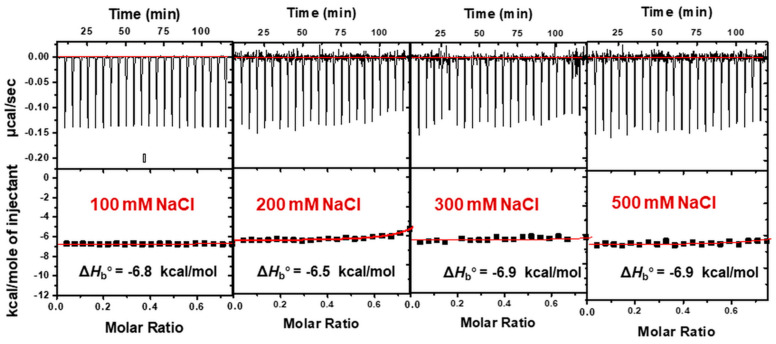
ITC data for the titration of DB2447 with AAAGTTT DNA at different salt concentrations at 25 °C. The listed binding enthalpies are an average of two independent experiments, and the values are reproducible within a 10% experimental error.

The ITC curves were fitted using Origin software to obtain the enthalpy at each titration point. The data (Figure 9) indicate an exothermic interaction after adding DB2447 to the solution containing -AAAGTTT-DNA at each NaCl concentration. The subtraction of the integrated peak areas for ligand/buffer titration from the ligand/DNA titration gives a direct determination of Δ*H*_b_° at each temperature. Figure 9 shows the titration of DB2447 into AAAGTTT with the blank buffer correction, and the Δ*H*_b_° value is −6.8 ± 0.2 kcal/mol at 25 °C at a 100 mM salt concentration. The favorable negative enthalpy change suggests strong H-bonding, electrostatic, and van der Waals interactions between DB2447 and AAAGTTT DNA. Interestingly, the complex formation of DB2447-AAAGTTT has an enthalpy at 25 °C that is larger than most of the A-tract minor groove binders reported in the literature [44,47,48,49]. This is expected from the known sequence-dependent differences in minor groove structure and hydration between the single G•C bp and all AT DNA sequences (Table 1).

To evaluate the relationship between thermodynamic measurements of the DB2447–AAAGTTT complex and experimental salt concentrations, the ITC experiments were carried out at 100–500 mM NaCl concentrations. The SPR results show that the binding constant decreases by almost ten times (Table 1) with increasing salt concentration. However, the ITC experiments show that salt concentrations have a much smaller effect on Δ*H*_b_° than on Δ*G*_b_° (Figure 9 and Table 1). This phenomenon reveals the enthalpy of complex formation for an energetic component, which is the sum of interactions such as hydrogen bond formation and van der Waals interactions and is essentially independent of salt concentration.

### 3.7. Determination of the Heat Capacity of the DB2447−AAAGTTT Complex

**Figure 10:**
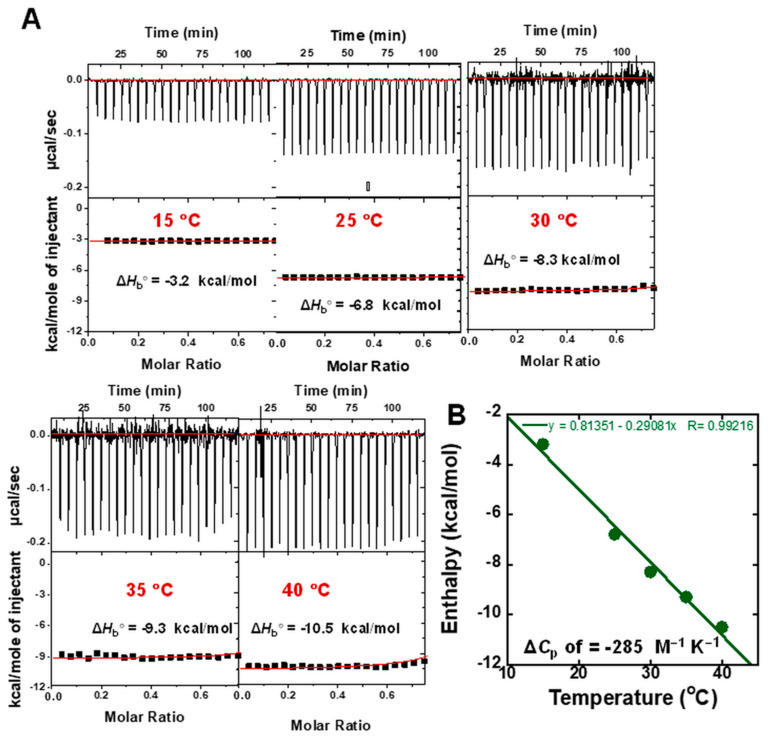
(**A**) ITC data for the titration of DB2447 with AAAGTTT DNA at different experimental temperatures by using 100 mM NaCl. (**B**) Plot of Δ*H*_b_° versus temperature for DB2447 with AAAGTTT DNA, and the linear fit yields a Δ*C*_p_ of −285 M^−1^ K^−1^. The listed binding enthalpies are an average of two independent experiments, and the values are reproducible within a 10% experimental error.

The ITC experiments of DB2447 with AAAGTTT were also carried out at different temperatures (15−40 °C) with a constant 100 mM NaCl concentration (Figure 10). The titration profiles indicate that the enthalpy of DB2447–DNA complex formation strongly depends on the experimental temperature and becomes more negative with increases in temperature (Table 2). The temperature-dependent differences in the binding enthalpy of DB2447–DNA complexes were used to calculate the heat capacity (Δ*C*_p_) for binding from the slope of a linear least-squares fit of the plot of Δ*H*_b_° versus temperature, Δ*C*_p_ = −285 cal/mol K (Figure 10). The temperature-dependent ITC results also show the effect of temperature on the entropy term. As the Δ*G*_b_° of binding is essentially constant with temperature (Figure 8, Table 2), the subtraction of Δ*H*_b_° from Δ*G*_b_° yields results that show that the entropy of binding decreases as the temperature is increased (Table 2) and, as previously noted, TΔ*S*_b_° approaches zero at 40–45 °C.

### 3.8. The Effects of AT Flanking Sequence Variations on DB2447 Binding Thermodynamics

**Figure 11:**
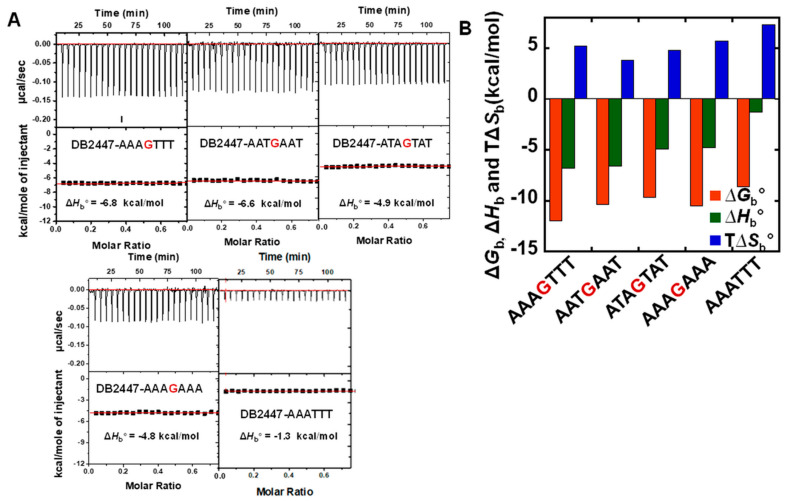
(**A**) ITC data for the titration of DB2447 with different single G•C base-pair-containing mixed DNA sequences in 50 mM Tris-HCl, 100 mM NaCl, 1 mM EDTA buffer, pH 7.4 at 25 °C. (**B**) Comparison of thermodynamic parameters, Δ*G*_b_°, Δ*H*_b_°, and TΔ*S*_b_°, of DB2447 with different single G•C base-pair-containing mixed DNA sequences. The plotted thermodynamic parameters are an average of two independent experiments, and the values are reproducible within a 10% experimental error. Full DNA sequences as described in Figure 2.

In Figure 11, the effects of the four sequences with different AT sequences flanking the single G•C bp binding site are shown. Determination of the ITC Δ*H*_b_° and the SPR Δ*G*_b_° (Figure 4) allows calculation of the Δ*S*_b_ for each sequence (Figure 11). The -AAAGTTT- sequence has the most favorable Δ*G*_b_° and with a substantial Δ*S*_b_°, which is why it is the best binding sequence. The AATGAAT sequence has a similar Δ*H*_b_° but a smaller Δ*S*_b_° with a decreased Δ*G*_b_°. With AAAGTTT and ATAGTAT, the binding Δ*H*_b_° is decreased but the Δ*S*_b_° is higher than with AATGAAT. With AAAGTTT, the binding becomes entropy-driven, and binding to AAATTT is shown as a strongly entropy-driven reference (Figure 11).

### 3.9. Thermodynamic Effects in the Binding of the Pyridyl Diamidine Compounds

**Figure 12:**
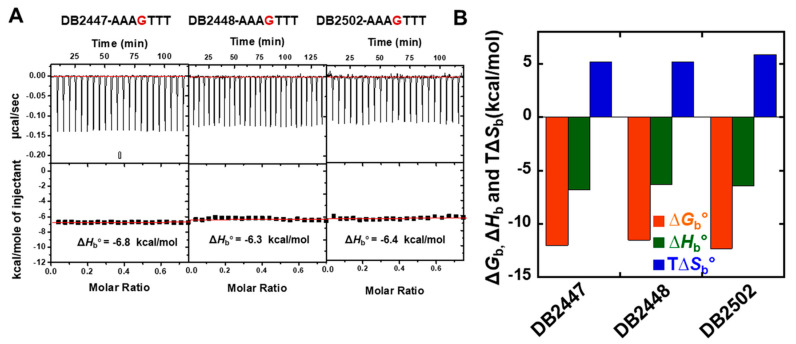
(**A**) ITC data for the titration of DB2447, DB2448, and DB2502 with AAAGTTT DNA sequence in 50 mM Tris-HCl, 100 mM NaCl, 1 mM EDTA buffer, pH 7.4 at 25 °C. (**B**) Comparison of thermodynamic parameters, Δ*G*_b_°, Δ*H*_b_°, and TΔ*S*_b_°, of DB2447, DB2448, and DB2502 with AAAGTTT. The plotted thermodynamic parameters are an average of two independent experiments, and the values are reproducible within a 10% experimental error.

The three compounds have similar thermodynamics with DB2502 having a slightly larger Δ*G*_b_°, while the value is smallest with DB2448 (Figure 12). DB2447 has the most favorable Δ*H*_b_°, suggesting that the unsubstituted amidines form the most favorable H-bonds. DB2502 has the most favorable Δ*S*_b_°, indicating that the large tetrahydropyrimidine displaces the most water from the minor groove on binding. 

### 3.10. Competition Electrospray Ionization Mass Spectrometry (ESI-MS) of DB2447

**Figure 13:**
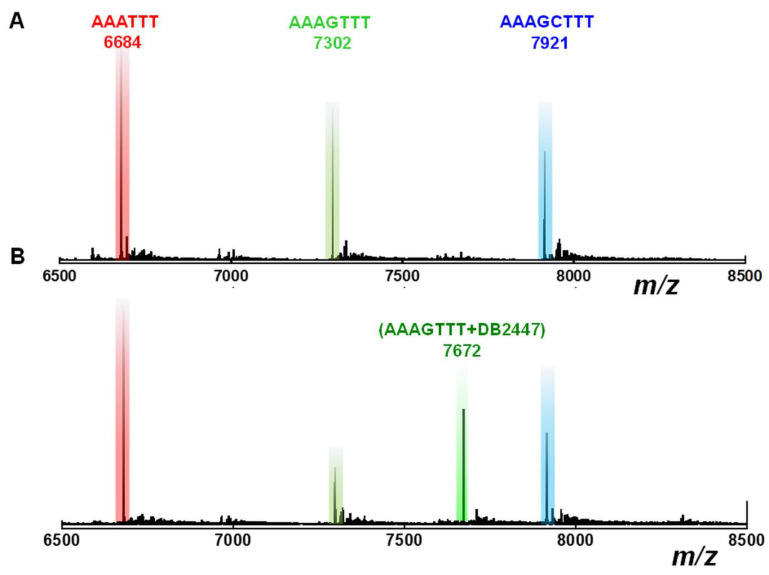
ESI-MS negative mode spectra of the competition binding of sequences AAATTT, AAAGTTT, and AAAGCTTT (10 µM each), with 30 µM DB2447 in buffer (100 mM ammonium acetate with 10% methanol (*v*/*v*), pH 6.8). (**A**) The ESI-MS spectra of free DNA mixtures; (**B**) the ESI-MS spectra of DNA mixtures with DB2447. The ESI-MS results are deconvoluted spectra, and molecular weights are shown with each peak.

Competition MS allows high-throughput screening for the comparison of binding of compounds to a panel of DNA sequences for the evaluation of relative affinity and selectivity [53,54]. The dashes represent zero, one, or two G•C bps with the AAA-TTT sequences used. In Figure 13A, the free DNA peaks are shown for AAATTT (*m*/*z* 6684), AAAGTTT (*m*/*z* 7302), and AAAGCTTT (*m*/*z* 7921). After the addition of DB2447, the intensity of the peak for AAAGTTT (*m*/*z* 7302) is reduced with the appearance of a new peak at *m*/*z* 7672, which is the characteristic of a 1:1 AAAGTTT–DB2447 complex (Figure 13B). This is in agreement with the Δ*T*_m_ and SPR results that show stronger binding to the single G•C bp sequence. At the 1:1 ratio of compound-to-DNA in this experiment, only binding to -AAAGTTT- is seen. There is no appearance of other DNA–ligand complex peaks at this compound-to-DNA ratio. As the ratio is increased beyond that in Figure 13, binding to other, less favored sequences begins to be observed. The observed ESI-MS spectra strongly indicate the high sequence specificity and affinity of DB2447 for the single G•C bp sequence.

## 4. Discussion

Over the last 50 years, there have been extensive studies on the interaction of a broad range of minor groove binders with DNA. Most of these compounds, especially those in the initial studies such as netropsin, DAPI, and Hoechst 33258 (Figure 1A), have been specific for binding to pure AT bp sequences, especially with A-tract type sequences. In the project described in this paper, the studies have been broadened to include new compounds from our laboratories that were designed to include a pyridine group with the goal to add a G•C bp to the traditional AT recognition sequence (Scheme 1, Figure 1C). The DNA sequences were also designed to include variations in the AT bp sequences that flank the G•C bp (Figure 3) to probe the effects of flanking sequences on affinity and specificity in binding. The pyridine group has been incorporated in the minor groove binders to complex with the G-NH that protrudes into the minor groove. One control compound has a phenyl in place of the pyridine to quantitatively evaluate the pyridine effects. The terminal cationic groups on the compounds (Figure 1) were varied to determine their role in DNA recognition. The compounds have either amidines, imidazolines, tetrahydropyridines, or, in one case, a combination of one amidine and one amide for a total of five compounds. The DNA samples included in the studies have zero, one, or two central GC base pairs with various flanking AT sequences (Figure 2). The combination gives five compounds and 12 DNA sequences for broad comparison with thermal melting, SPR, ITC, and combination MS methods under a variety of salt concentration and temperature conditions.

The Δ*T*_m_ results present a complete but low-resolution picture of all five compounds with the 12 DNA samples. For all of the pyridine derivatives, the single GC sequences have the highest Δ*T*_m_ values, and the highest of all is obtained with the -AAAGTTT- sequence (Figure 4, Table S1). It clearly has the optimum combination of groove width, curvature, and placement of H-bond acceptor groups in the minor groove to interact with the pyridine-substituted compounds (Figure 1C). With DB2447, for example, the Δ*T*_m_ with -AAAGTTT- is 14 °C, while the Δ*T*_m_ values for all other single G DNAs are between 5 and 7, about one-half the -AAAGTTT- Δ*T*_m_. In all cases, the alternating AT sequence -ATAGTAT- has the lowest Δ*T*_m_ values with all DNA sequences. This sequence has a significantly wider minor groove width than -AATGTTT- and is a less favorable binding site for these types of minor groove binders (Figure 3). DB2448, with imidazoline cationic groups, binds a little weaker than the amidine, but it has excellent selectivity and only has a significant Δ*T*_m_ with single G•C sequences. DB2502 with tetrahydropyrimidine terminal cations binds more strongly than the other two compounds but with lower specificity. Ab initio calculations on the three pyridine dications indicate that DB2448 is the most planar structure while DB2502 is the least planar. DB2559 with the pyridine replaced with a phenyl binds best to pure AT sequences as expected. It has the best binding with -AAATTT-, while all other pure AT sequences only have about one-half of the Δ*T*_m_ of -AAATTT-.

Biosensor-SPR methods provide more quantitative binding results than the Δ*T*_m_ values but they are in qualitative agreement (Figure 4 and Figure 5). The *K*_D_ for DB2447 with -AAAGTTT- is 1.8 nM, and results with -AAAGAAA- and AATGAAT are ten-fold higher, in agreement with their lower Δ*T*_m_ values. As with Δ*T*_m_, the weakest binding is seen with -ATAGTAT- with a *K*_D_ of 68 nM. The same trend is observed with the other pyridine compounds with the Δ*T*_m_ results: DB2448 binds slightly weaker but with greater selectivity, while DB2502 binds slightly more strongly but with lower selectivity. DB2559 binds best to pure AT sequences and, as expected, it has the strongest binding to -AAATTT-.

Given that -AAAGTTT- is the best binding sequence, more detailed thermodynamic studies were conducted with it and DB2447 to determine what components are most important for complex formation. As is typical of many biological complexes that are formed from numerous relatively weak interactions, the pyridine DB2447 complex in this set has a large negative heat capacity for complex formation, −248 cal/mol deg. With DB2447, the Δ*H*_b_° and −TΔ*S*_b_ values are −6.8 and −5.1 kcal/mol at 25 °C, respectively. As expected from the negative heat capacity, the values at 40 °C are −11.4 and −0.90 kcal/mol and the compensating changes maintain an almost constant Δ*G*_b_°. This agrees with the thermodynamics for other minor groove binders that interact with a G•C bp and indicates an enthalpy-driven complex stabilized by an array of H-bonding, van der Waals, and electrostatic interactions [22,55]. With compounds such as DB75 that recognize only AT bp sequences, the binding entropy is the dominant component of the complex formation due to the release of water from the minor groove AT sites [49]. With DB2559, for example, the Δ*H*_b_° and −TΔ*S*_b_° values are approximately −4 (Figure S6) and −6 kcal/mol at 25 °C, respectively, and the difference is even larger with DB75, approximately −2 and −7 kcal/mol, respectively [49]. The clear conclusion from the available results then is that adding a G•C bp to a minor groove binder recognition sequence significantly increases the binding enthalpy and reduces the binding entropy. A similar thermodynamic shift was seen with DB293 and DB2277 [22,55]. With an -AATT- sequence, however, DB293 binds as an entropy-driven complex [56]. In all of these systems, the binding energetics shift more to energetic emphasis on H-bond formation through the G-NH to compound acceptor group and more to water release and entropy in a pure AT minor groove sequence.

**Figure 14:**
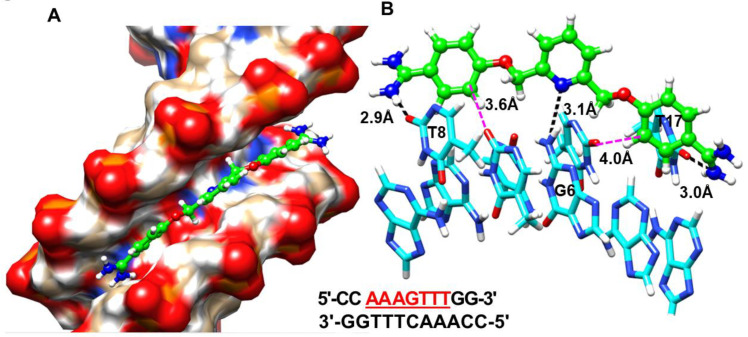
(**A**) A Snapshot of minor groove view of the DB2447-d[(5′-CCAAAGTTTGG-3′)(5′-CCAAACTTTGG-3′)] complex from MD simulations. The sphere model in a green-white-blue-red-yellow (C-H-N-O-S) color scheme represents DB2447. The DNA bases are represented in a ball and stick and ribbon model with a cyan-white-red-blue-orange (C-H-O-N-P) color scheme. (**B**) The important interactions between different sections of the DB2447–DNA complex are illustrated. DB2447 forms three direct H-bonds (black dashed lines) with DNA bases. Red lines indicate compound aromatic -CH interactions with -T=O groups.

Strong support for the highly selective binding of DB2447 to -AAAGTTT- is seen in competition mass spectroscopy experiments (Figure 13). This is in agreement with the Δ*T*_m_ and SPR results that show stronger binding to the single G•C sequence. To help better understand the structural basis of molecular recognition of DNA sequences with a GC bp in an AT flanking sequence context, a molecular dynamics (MD) simulation for a complex of the pyridine compound, DB2447, with the DNA sequence *ds*[5′-CCAAAGTTTGG-3′)(5′CCAAACTTTGG-3′)] was conducted (Figure 14). Force constants for DB2447 were determined as described previously and added to the force field for the simulation [57,58]. The MD simulation was performed by using Amber 16 in the presence of 0.15 M NaCl as previously described. The DB2447 complex can dynamically orient to provide favorable curvature to the DNA complex and interactions between the compound and DNA. The pyridine N and amidine -NH groups are positioned for strong H-bonds with the -G-NH (3.1 A) and -two T=O (2.9–3.0 A) groups at the floor of the minor groove (Figure 14). The H-bonding ability, stacking with the minor groove walls, and dynamics of the bound system help provide the high binding affinity of DB2447 to the -AAAGTTT- binding site. The strong G-NH to pyridine N H-bond provides high binding selectivity of DB2447 toward the AAAGTTT sequence, in agreement with the ESI-MS results. Additional selectivity in binding is provided by −CH groups of the two phenyls that point into the minor groove (Figure 14B). The −CH groups form a dynamic weak interaction with −dT=O that are adjacent to the central G·C bp. The combination of weak to strong interactions in the complex gives the large negative heat capacity and strong binding of DB2447.

For drug design, it is essential to understand the effects of solution conditions on minor groove binder–DNA complexes. The equilibrium constant, *K*_A_, decreases as the salt concentration is increased as with other minor groove binders. As expected, the slope of a log*K*_A_ versus log[Na^+^] plot is linear with a slope of 1.6 (Figure 6). The enthalpy change, Δ*H*_b_°, is affected by a very small amount with changes in salt concentration. The effects of salt concentration on Δ*G*_b_° and TΔ*S*_b_° are complementary and amount to about a one-kilocalorie decrease as the salt concentration increases from 100 to 500 mM NaCl. As the temperature increases, however, Δ*H*_b_° becomes much more negative and the heat capacity for binding is a large negative value for binding a small molecule.

ITC experiments were also used to evaluate the effects of the DNA sequence on the binding of DB2447. The results show (Figure 11) that the -AAAGTTT- sequence has substantial Δ*H*_b_° and Δ*S*_b_ values and the most favorable Δ*G*_b_°, which is why it is the best binding sequence. The -ATAGATA- sequence has a relatively small Δ*H*_b_° and Δ*S*_b_ that sum to give it the lowest Δ*G*_b_° value of all the single G sequences. Evaluation of the three pyrimidine diamidine compounds shows that they all have similar binding thermodynamics. DB2502 has the most favorable Δ*S*_b_, indicating that the cyclic tetrahydropyrimidine group displaces the most water from the minor groove on binding. DB2448 has the lowest Δ*S*_b_ that sums with its Δ*H*_b_° to give it the lowest Δ*G*_b_° of the three compounds.

In summary, the results presented here show that minor groove binding thermodynamics depends on both compound structure and DNA sequence. 

## Figures and Tables

**Table 1:** Kinetics ^a^ and steady-state ^b^ analysis of DB2447 with the AAAGTTT sequence at different salt concentrations in 50 mM Tris-HCl, 1 mM EDTA buffer, pH 7.4 at 25 °C.

NaCl (mM)	*K*_D_ (nM)	Δ*G*_b_° (kcal/mol)	Δ*H*_b_° (kcal/mol)	TΔ*S*_b_° (kcal/mol)
100	1.4	−12.0	−6.8	5.2
200	4.3	−11.4	−6.5	4.9
300	7.8	−11.1	−6.9	4.2
500	15.8	−10.7	−6.9	3.8

^a^ Kinetics analysis was performed by global fitting with a 1:1 binding model and ^b^ steady-state fits were carried out by using 1:1 binding equation model. Δ*H*_b_° was determined in ITC experiments; TΔ*S*_b_ = −Δ*G*_b_° + Δ*H*_b_°.

**Table 2:** Kinetics and thermodynamic results for DB2447 with the AAAGTTT sequence at different experimental temperatures at 50 mM Tris-HCl, 100 mM NaCl, 1 mM EDTA buffer at pH 7.4.

Temperature (°C)	*K*_D_ (nM)	Δ*G*_b_° (kcal/mol)	Δ*H*_b_° (kcal/mol)	TΔ*S*_b_° (kcal/mol)
25	1.4	−12.0	−6.8	5.2
30	2.0	−11.8	−8.3	3.5
35	2.5	−11.7	−9.3	2.4
40	4.2	−11.4	−10.5	0.9

## Data Availability

Not applicable.

## Supplementary Materials

The following supporting information can be downloaded at: https://www.mdpi.com/article/10.3390/life12050681/s1. References [21,22,24,28] are cited in the supplementary materials. Experimental details of synthesis, DNA melting experimental data table, SPR sensorgrams, and affinity binding curves for DB2447, DB2448, DB2449, DB2502, and DB2559; SPR equilibrium dissociation constants (*K*_D_, nM) data table for DB2447 and analogs with pure A·T and mixed DNA sequences; ITC of DB2559 and DB2448 with AAATTT sequence; ^1^H NMR spectra of final products. Figure S1: Comparison of equilibrium binding constants (*K*_D_, M) of DB2447 with pure AT and mixed single/two G•C base pair(s) containing DNA sequences; Figure S2: Comparison of equilibrium binding constants (*K*_D_, M) of DB2559 with pure AT and mixed single G•C base-pair containing DNA sequences; Figure S3: Comparison of equilibrium binding constants (*K*_D_, M) of DB2448 with mixed single/two G•C base pair(s) containing DNA sequences; Figure S4: Comparison of equilibrium binding constants (*K*_D_, M) of DB2502 with pure AT and single/two G•C base pair(s) containing DNA sequences; Figure S5: Comparison of equilibrium binding constants (*K*_D_, M) of DB2449 with mixed single/two G•C base pair(s) containing DNA sequences; Table S1: Thermal Melting Studies (Δ*T*_m_, ^a^ °C) of the designed heterocyclic amidine compounds with pure A·T and mixed DNA sequences; Table S2: Biosensor-SPR equilibrium dissociation constants (*K*_D_, nM) of DB2447 and analogues with pure A·T and mixed DNA sequences; Figure S6: ITC data for the titration of DB559 and DB2448 with AAATTT DNA at 100 mM NaCl at 25 °C.
