# Supplementary material for: Thermodynamic Factors That Drive Sequence-Specific DNA Binding of Designed, Synthetic Minor Groove Binding Agents
Source: Life (Basel). 2022 May 4;12(5):681. doi: 10.3390/life12050681 (PMC9147024; doi:10.3390/life12050681)
Supplement: Supplementary file 1 [file life-12-00681-s001.zip › life-1683280-supplementary.pdf]

Supporting Information

Thermodynamic Factors that Drive Sequence-Specific DNA Binding of Designed, Synthetic Minor Groove Binding Agents

Ananya Paul[1], Abdelbasset A Farahat[1,2], David W. Boykin[1], and W. David Wilson[1,*]

[1]Department of Chemistry and Center for Diagnostics and Therapeutics, Georgia State University, Atlanta, GA 30303, USA

[2]Department of Pharmaceutical Organic Chemistry, Faculty of Pharmacy, Mansoura University, Mansoura 35516, Egypt

Address correspondence to this author

*W. David Wilson Tel: 404-413-5503; Fax: 404-413-5505; Email: wdw@gsu.edu

EXPERIMENTAL METHODS

Synthesis:

4,4'-((Pyridine-2,6-diylbis(methylene))bis(oxy))dibenzimidamide (DB 2447)

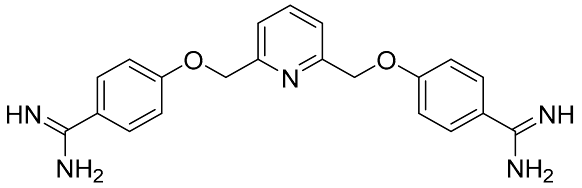

White solid, yield (0.51 g, 68%), mp > 300 °C, ^{1}HNMR (DMSO-d_6) δ 9.30 (s, 4H), 9.09 (s, 4H), 7.94 (t, *J* = 8 Hz, 1H), 7.88 (d, *J* = 8.8 Hz, 4H), 7.53 (d, *J* = 8. 2H), 7.27 (d, *J* = 8.8 Hz, 4H), 5.34 (s, 4H) ; ESI-MS: m/z calculated for $C_{21}H_{22}N_5O_2$: 376.1777, found: 376.1768 (amidine base M^++1); Anal. Calcd. For $C_{21}H_{21}N_5O_2$. 3HCl. $1H_2O$: C, 50.16; H, 5.21; N, 13.92. Found: C, 50.01; H, 5.13; N, 13.77.

2,6-bis((4-(4,5-dihydro-1H-imidazol-2-yl)phenoxy)methyl)pyridine (DB 2448).

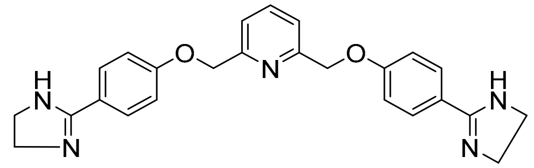

White solid, yield (0.54 g, 66%), mp > 300 °C, ^{1}HNMR (DMSO-d_6) δ 10.74 (s, 4H), 8.12 (d, *J* = 8.8 Hz, 4H) 7.94 (t, *J* = 8 Hz, 1H), 7.54 (d, *J* = 8 Hz. 2H), 7.29 (d, *J* = 8.8 Hz, 4H), 5.35 (s, 4H), 3.96 (s, 8H); ESI-MS: m/z calculated for $C_{25}H_{26}N_5O_2$: 428.2091, found: 428.2081 (amidine base M^++1); Anal. Calcd. For $C_{25}H_{25}N_5O_2$. 3HCl. $1H_2O$: C, 54.11; H, 5.44; N, 12.62. Found: C, 54.09; H, 5.38; N, 12.57.

4-((6-((4-(amino(l4-azaneylidene)methyl)phenoxy)methyl)pyridin-2-yl)methoxy)benzamide (DB 2449).

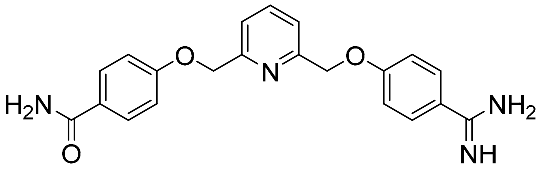

White solid, yield (0.51 g, 69%), mp > 300 °C, ^{1}HNMR (DMSO-d_6) δ 9.26 (s, 2H), 9.05 (s, 2H), 7.94 (t, *J* = 8 Hz, 1H), 7.86 (d, *J* = 8.8 Hz, 4H), 7.53 (d, *J* = 8. 2H), 7.27 (d, *J* = 8.8 Hz, 2H), 7.08 (d, *J* = 8.8 Hz, 2H), 5.35 (s, 2H), 5.27 (s, 2H); ESI-MS: m/z calculated for $C_{21}H_{21}N_4O_3$: 377.1619, found: 377.1608 (amidine base M^++1); Anal. Calcd. For $C_{21}H_{20}N_4O_3$. 2HCl. 2.5H_2O: C, 51.01; H, 5.50; N, 11.33. Found: C, 50.91; H, 5.62; N, 11.22.

2,6-bis((4-(1,4,5,6-tetrahydropyrimidin-2-yl)phenoxy)methyl)pyridine (DB 2502).

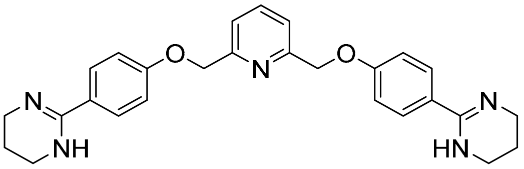

White solid, yield (0.6 g, 69%), mp > 300 °C, ^{1}HNMR (DMSO-d_6) δ 9.98 (s, 4H), 7.93 (t, *J* = 8 Hz, 1H), 7.78 (d, *J* = 8.8 Hz, 4H), 7.52 (d, *J* = 8 Hz. 2H), 7.26 (d, *J* = 8.8 Hz, 4H), 5.32 (s, 4H), 3.47 (br s, 8H), 1.95 (t, *J* = 4.8 Hz, 4H); ESI-MS: m/z calculated for $C_{27}H_{30}N_5O_2$: 456.2407, found: 4546.2394 (amidine base M^++1); Anal. Calcd. For $C_{27}H_{29}N_5O_2$. 3HCl. 1.25H_2O: C, 55.20; H, 5.91; N, 11.92. Found: C, 55.18; H, 5.86; N, 11.68.

Materials and Methods

Ligand -DNA binding biophysical experimental materials, methods of Uv-vis DNA melting, Biosensor Surface-plasmon Resonance (SPR), Isothermal Titration Calorimetry (ITC), Competition Electrospray Ionization Mass Spectrometry (ESIMS), and Molecular dynamic simulation procedures have been reported in previous publications.[1-4]

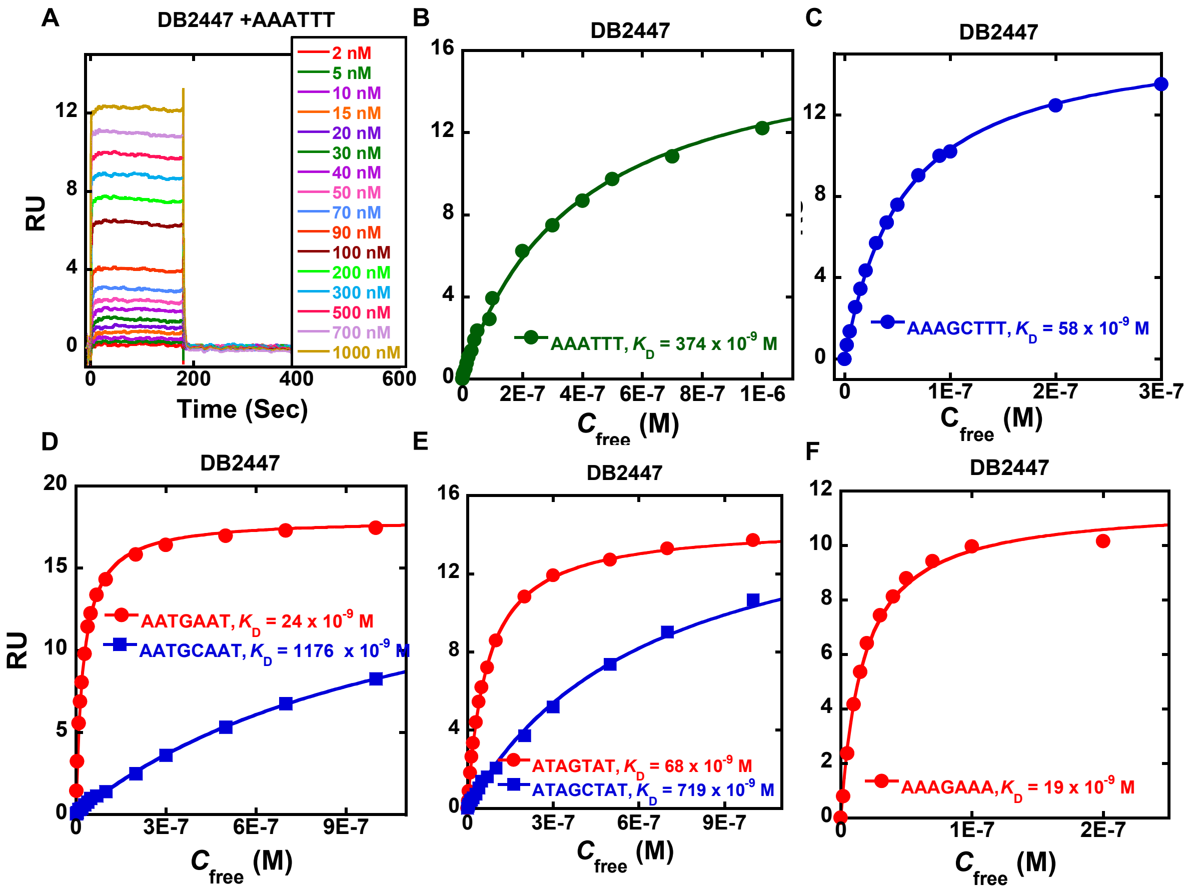

Figure S1. Comparison of equilibrium binding constants (K_D, M) of DB2447 with pure AT AAATTT (A, B) and mixed single/two G•C base pair(s) containing (AAAGCTTT (C), AATGAAT (D), AATGCAAT (D), ATAGTAT (E), ATAGCTAT (E), and AAAGAAA (F)) DNA sequences. The listed binding affinities are an average of two independent experiments carried out with two different sensor chips and the values are reproducible within 10% experimental error. Full DNA sequences as described in Figure 2.

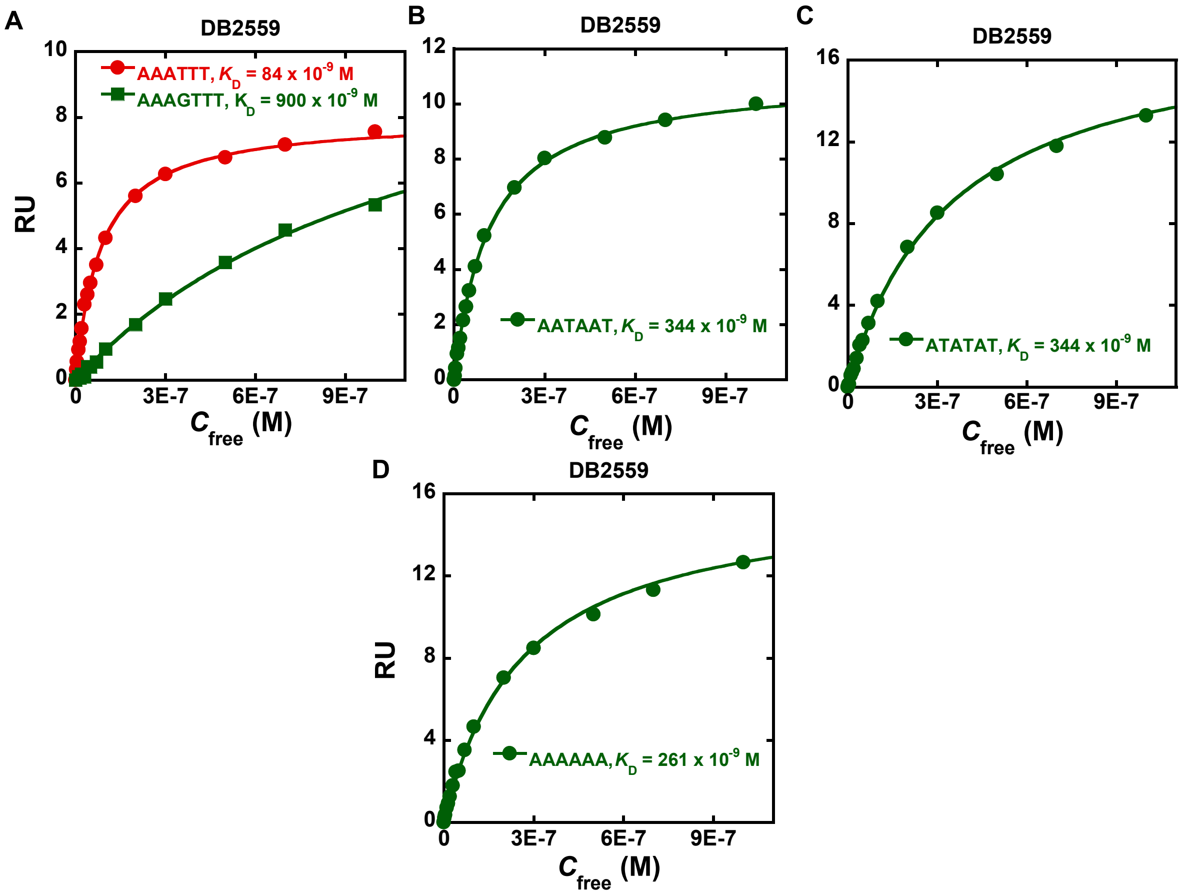

Figure S2. Comparison of equilibrium binding constants (K_D, M) of DB2559 with pure AT (AAATTT (A), AATAAT (B), ATATAT (C) and AAAAAA (D)) and mixed single G•C base-pair containing (AAAGTTT (A)) DNA sequences. The listed binding affinities are an average of two independent experiments carried out with two different sensor chips, and the values are reproducible within 10% experimental error. Full DNA sequences as described in Figure 2.

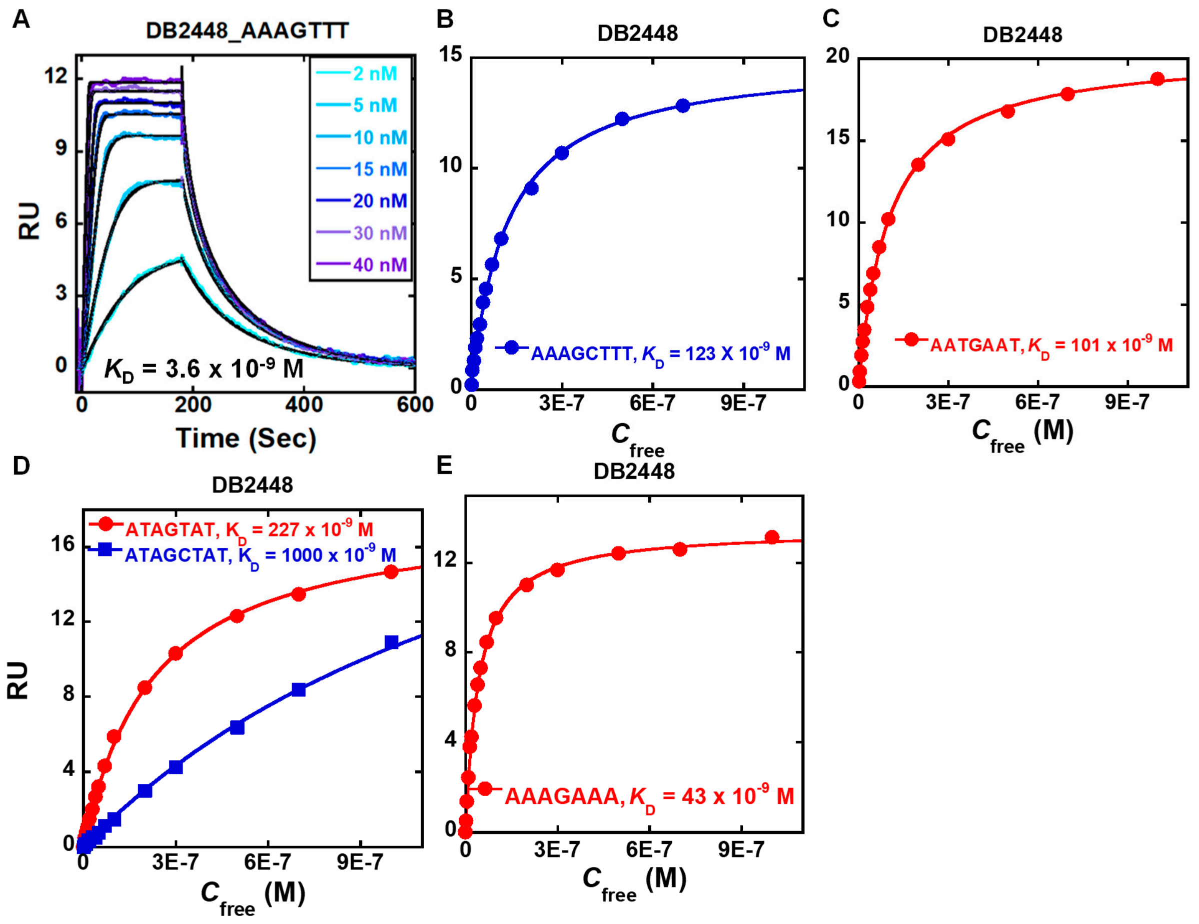

Figure S3. Comparison of equilibrium binding constants (K_D, M) of DB2448 with mixed single/two G•C base pair(s) containing (AAAGTTT (A), AAAGCTTT (B), AATGAAT (C), ATAGTAT (D), ATAGCTAT (D), and AAAGAAA (E)) DNA sequences.The listed binding affinities are an average of two independent experiments carried out with two different sensor chips, and the values are reproducible within 10% experimental error. Full DNA sequences as described in Figure 2.

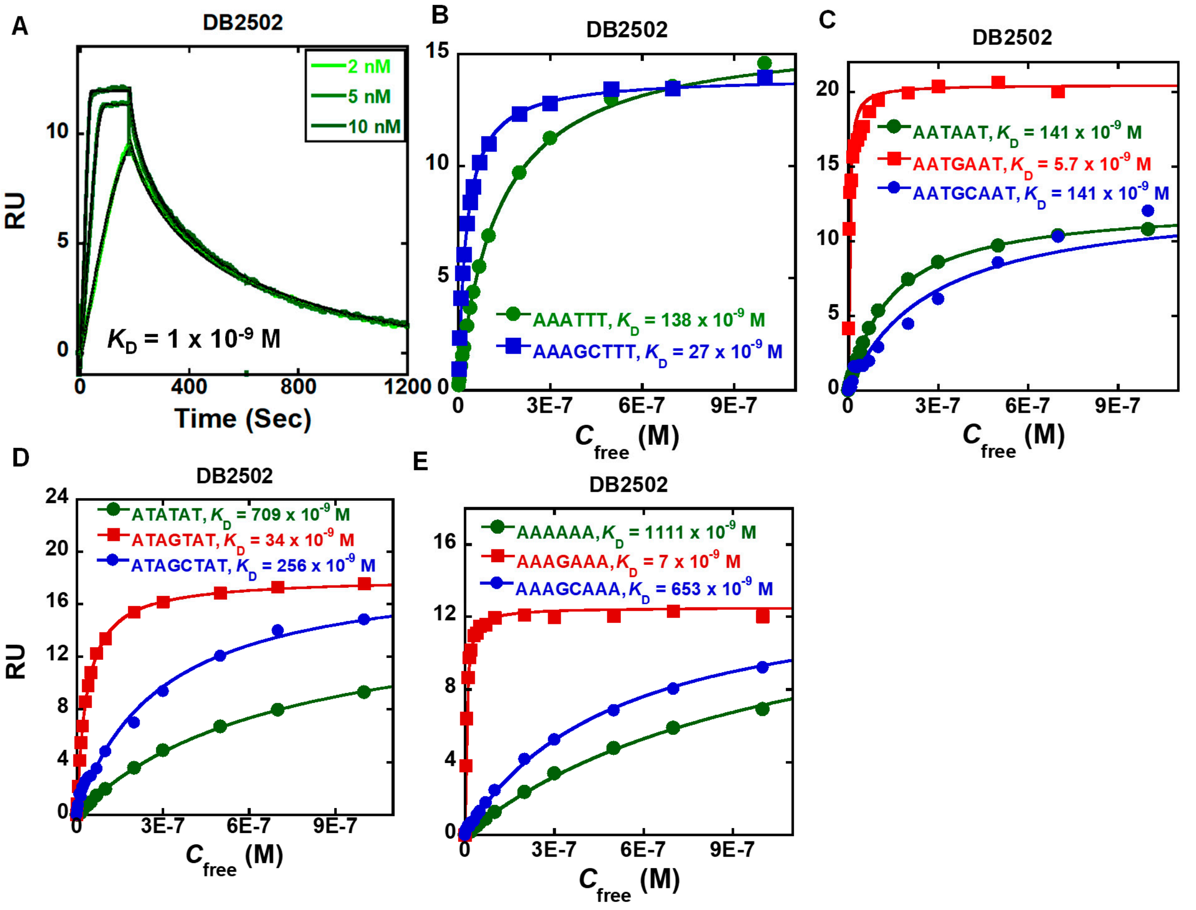

Figure S4. Comparison of equilibrium binding constants (K_D, M) of DB2502 with pure AT (AAATTT (B), AATAAT (C), ATATAT (D), and AAAAAA (E)) and single/two G•C base pair(s) containing (AAAGTTT (A), AAAGCTTT (B), AATGAAT (C), AATGCAAT (D), ATAGTAT (D), ATAGCTAT (D), AAAGAAA (E), and AAAGCAAA (E)) DNA sequences. The listed binding affinities are an average of two independent experiments carried out with two different sensor chips and the values are reproducible within 10% experimental errors. Full DNA sequences as described in Figure 2.

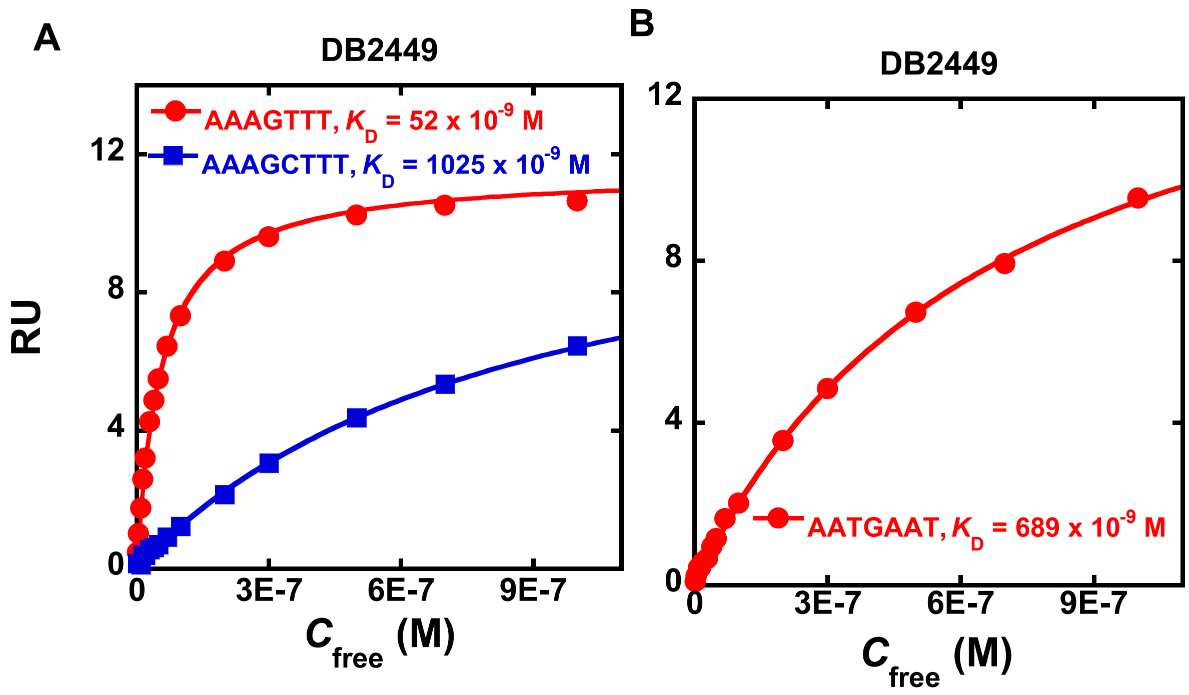

Figure S5. Comparison of equilibrium binding constants (K_D, M) of DB2449 with mixed single/two G•C base pair(s) containing (AAAGTTT (A), AAAGCTTT (A), and AATGAAT (B)) DNA sequences. The listed binding affinities are an average of two independent experiments carried out with two different sensor chips, and the values are reproducible within 10% experimental error. Full DNA sequences as described in Figure 2.

Table S1: Thermal Melting Studies (ΔT_m, [a] °C) of the Designed Heterocyclic Amidine Compounds with Pure A·T and Mixed DNA Sequences

DNA	DB2447 (ΔT_m °C)	DB2448 (ΔT_m °C)	DB2449 (ΔT_m °C)	DB2502 (ΔT_m °C)	DB2559 (ΔT_m °C)
**AAATTT**	**5**	**<1**	**2**	**7**	**9**
**AAAGTTT**	**14**	**11**	**4**	**15**	**4**
**AAAGCTTT**	**5**	**<1**	**1**	**7**	**<1**
**AATGAAT**	**7**	**5**	**1**	**10**	**1**
**AATGCAAT**	**2**	**1**	**1**	**2**	**1**
**ATAGTAT**	**5**	**3**	**<1**	**7**	**<1**
**ATAGCTAT**	**2**	**<1**	**<1**	**2**	**<1**
**ATATAT**	**<1**	**<1**	**<1**	**2**	**4**
**AAAAAA**	**<1**	**<1**	**<1**	**2**	**3**
**AAAGAAA**	**6**	**5**	**<1**	**11**	**<1**
**AAAGCAAA**	**1**	**<1**	**<1**	**2**	**<1**

[a] :$\Delta T_m = T_m$ (the complex) − T_m (the free DNA). The listed values are for 2:1 [ligand]/[DNA] ratio and an average of two independent experiments with reproducibility of ±0.5 °C

Table S2: Biosensor-SPR Equilibrium Dissociation Constants (K_D, nM) of DB2447 and Analogues with Pure A·T and Mixed DNA Sequences[a]

DNA	DB2447 (K_D nM)	DB2448 (K_D nM)	DB2449 (K_D nM)	DB2502 (K_D nM)	DB2559 (K_D nM)
**AAATTT**	**374**	**NB**	**NB**	**138**	**84**
**AAAGTTT**	**1.4**	**3.6**	**52**	**1**	**900**
**AAAGCTTT**	**58**	**123**	**1025**	**27**	**NB**
**AATAAT**	**NB**	**NB**	**NB**	**141**	**117**
**AATGAAT**	**24**	**101**	**689**	**5.7**	**NB**
**AATGCAAT**	**1176**	**NB**	**NB**	**534**	**NB**
**ATATAT**	**NB**	**NB**	**NB**	**709**	**344**
**ATAGTAT**	**68**	**227**	**NB**	**33.6**	**NB**
**ATAGCTAT**	**719**	**1000**	**NB**	**256**	**NB**
**AAAAAA**	**NB**	**NB**	**NB**	**1111**	**261**
**AAAGAAA**	**19**	**43**	**NB**	**4.6**	**NB**
**AAAGCAAA**	**NB**	**NB**	**NB**	**653**	**NB**

[a] The listed binding affinities are an average of two independent experiments carried out with two different sensor chips, and the values are reproducible within 10% experimental errors. The experiments were conducted in Tris-HCl buffer (50 mM Tris-HCl, 100 mM NaCl, 1 mM EDTA, pH 7.4) at 25 °C.

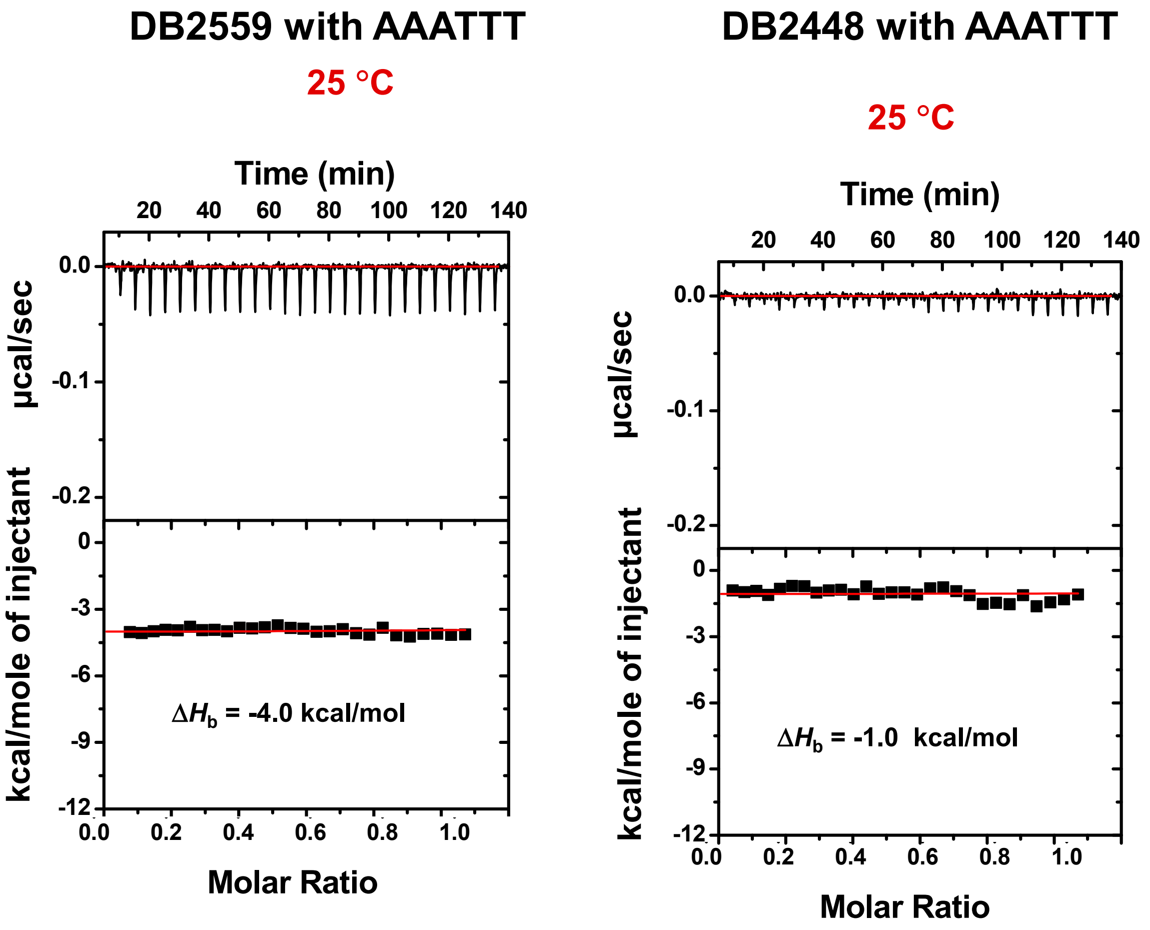

Figure S6. ITC data for the titration of DB559 and DB2448 with AAATTT DNA at 100 mM NaCl at 25 °C. The listed binding enthalpies are an average of two independent experiments, and the values are reproducible within 10% experimental errors.

References

1. Chai, Y.; Paul, A.; Rettig, M.; Wilson, W.D.; Boykin, D.W. Design and synthesis of heterocyclic cations for specific DNA recognition: from AT-rich to mixed-base-pair DNA sequences. *J. Org. Chem*. **2014**, *79*, 852-866.
2. Paul, A.; Chai, Y.; Boykin, D.W.; Wilson, W.D. Understanding mixed sequence DNA recognition by novel designed compounds: the kinetic and thermodynamic behavior of azabenzimidazole diamidines. *Biochemistry* **2015**, *54*, 577-587.
3. Paul, A.; Kumar, A.; Nanjunda, R.; Farahat, A.A.; Boykin, D.W.; Wilson, W.D. Systematic synthetic and biophysical development of mixed sequence DNA binding agents. *Org. Biomol. Chem*. **2017**, 15, 827-835.
4. Guo, P.; Farahat, A.A.; Paul, A.; Boykin, D.W.; Wilson, W.D. Engineered modular heterocyclic-diamidines for sequence-specific recognition of mixed AT/GC base pairs at the DNA minor groove. *Chem. Sci*. **2021**, 12,15849-15861
